# Comparing food consumption during the COVID-19 pandemic: analysis of household income and expenditure survey data in Iran

**DOI:** 10.1186/s41043-023-00385-3

**Published:** 2023-05-17

**Authors:** Mojtaba Hajipoor, Pegah Rahbarinejad, Kiyavash Irankhah, Seyyed Reza Sobhani

**Affiliations:** 1 Department of Nutrition Sciences, Varastegan Institute for Medical Sciences, Mashhad, Iran; 2 Department of Nutrition, School of Medicine, Mashhad University of Medical Sciences, Mashhad, Iran; 3 Student Research Committee, Mashhad University of Medical Sciences, Mashhad, Iran; 4 Department of Nutrition, Faculty of Medicine, Mashhad University of Medical Sciences, Mashhad, Iran

**Keywords:** Food consumption, COVID-19, Household income and expenditure data, Iran

## Abstract

**Background:**

On March 11, 2020, the WHO declared the outbreak of the infectious disease COVID-19 as a pandemic. The health strategies of nations lead to possible changes in lifestyle and increase poor eating habits. Hence, the purpose of this study is to compare food consumption during COVID-19 pandemic in Iran.

**Methods:**

This cross-sectional study used secondary data from the Households Income and Expenditure Survey (HIES) conducted annually by the Statistical Centre of Iran. Food cost data of HIES included the amount of all food items in household food baskets during the last month. Then, they were classified into six food groups to evaluate their energy intake. The consequence of food consumption was analyzed as a function of socioeconomic status (SES) variables and residence pre- and post-COVID-19 pandemic.

**Results:**

In total, 75,885 households (83.5% male) were included in the study. Among the population of urban and rural areas as well as in different SES categories, people tended to increase the consumption of meat (*P* < 0.05) and fresh foods, especially vegetable groups (*P* < 0.001) and decrease the consumption of fruit (*P* < 0.001), fat and sweets groups (*P* < 0.05) and also in energy intake (*P* < 0.05). Macronutrient changes were different in the category of SES, urban and rural.

**Conclusion:**

Our study indicated that the COVID-19 pandemic had different effects on food groups, energy and macronutrients consumption, which could be due to possible changes in food patterns as a result of the pandemic.

## Introduction

In December 2019, an infectious disease of deadly pneumonia instances of then unknown etiology in Wuhan, China, alerted the scientific and clinical communities [1]. The causal agent, caused by severe acute respiratory syndrome coronavirus 2 (SARS-CoV-2), spread unexpectedly across the world. As of January 16, 2021, SARS-CoV-2 caused nearly 2.1 million deaths with more than 93.8 million infections in 218 countries; therefore, the WHO Emergency Committee declared a pandemic state in March 2020 [2, 3].

Health strategies of nations to prevent the spread of coronavirus without effective and safe vaccines included quarantine and social distancing [4]. Control measures (including staying at home, social distancing, and closing public places) are very effective in reducing the spread of infections. However, this control measures limited access to vital services [3].

Restrictions during the pandemic changed the lifestyle of communities and had major impacts on physical and mental health, as well as on social and economic aspects [5]. Also, the implementation of health strategies induced restrictions on the movement of workers, roads, and food production, on the other hand, the increase in consumer demand caused a decrease in available food [6]. A possible consequence of restrictions is a change in lifestyle and eating habits that cause changes in food intake. This change is due to the reduced availability of goods, limited working hours of stores, and a switch to unhealthy food [7]. A current study on the psychological impact of quarantine reported negative psychological effects such as post-traumatic stress, confusion, and anger [8]. The psychological effects of changing food choices could have led to irregular eating and more frequent snacking [9, 10].

During the restrictions and quarantine, the disruption in the production system (due to decreased the number of workers and work capacity) and in the consumer system (due to reduced demand and lack of purchasing power due to reduced employment and income), caused problems in the food and nutrition system [11]. On the other hand, consumer concerns about food supply can be associated with increased food demand such as food hoarding and panic buying, which can have devastating consequences on diet quality and malnutrition [12, 13]. The effects of this consequence include increasing the consumption of low-calorie foods (such as starchy foods and processed and unhealthy grains), reducing food rich in nutrients, and reducing the number of meals, which lead to insufficient food intake and an increase in undernutrition or overweight/obesity [14].

According to a recent study conducted in the world, it was found that the environment created by the COVID-19 pandemic had adverse effects on physical activity, diet, and sleep [15]. The consumption of fresh food (including fruits and vegetables) was reduced due to reduced shopping frequency. In contrast, the consumption of food with a longer shelf life (including frozen foods, canned foods, and sweets) increased. Also, bread and dairy products were with the lowest rate of change in consumption, but studies on alcohol were conflicting [16, 17]. In addition, it has been seen unhealthy diets and reduced physical activity could negatively affect health status and lead to obesity [5, 18] and risk of cardiovascular disease [18] Which, in turn, relates to an increased risk of mortality among patients with COVID-19 [19, 20].

In Iran, studies reported significant changes in dietary habits and consumption of supplements due to the COVID-19 pandemic. These dietary changes included consuming more fruits, vegetables, rice, bread, and nutritional supplements and less fish, dairy, and fast foods, but studies were conflicting on meat and protein-rich foods [21–23]. To resolve this controversy in the studies, our study used secondary data from the Households Income and Expenditure Survey (HIES) of the Statistical Centre of Iran (SCI) (including 75,885 populations, as a large sample size) to identify the possible effects of COVID-19 on household food intake through different mechanisms. In addition, coverage of energy and macronutrient intake can make the distinction between this study and other studies. To the best of our knowledge, there was no study to compare food consumption before and after the COVID-19 pandemic in Iranian households, therefore the present study aimed to evaluate changes in food intake at two time points in Iran.

## Methods

### Participants

This cross-sectional study used secondary data from the Households Income and Expenditure Survey (HIES) that is yearly carried out by the Statistical Centre of Iran (SCI). There were 38,328 respondents at the national level in 2019 (before the COVID-19 pandemic) and 37,557 respondents in 2020 (after the COVID-19 pandemic). All private and collective settled households in urban and rural areas are the target population of HIES. A three-stage cluster sampling method with strata was used in the survey. The samples were equally distributed between the months of the year to obtain estimations more representative of the whole year. The HIES, which aims to estimate the average income and expenditure for urban and rural households, included demographics, consumable (e.g., expenditures on foods) and nonconsumable costs, and household income information. Available socioeconomic variables in HIES data were as follow: (1) household’s head variables: age, gender (male/female), and occupational category status; (2) household variables: education level, living area (rural/urban), annual income, house area, and size of the family (number of household members) [24].

All participants gave written informed consent and provided assent to participate. The protocols of this study were approved by the Research Ethics Committees of school of Medicine affiliated with the Mashhad University of Medical Sciences. The approval ID of the present study was IR.MUMS.MEDICAL.REC.1400.404.

### Food data

HIES collected comprehensive data on the total amount of food purchased during the previous month, as well as food items received as gifts or donations, and any food produced by household members. Data from household food baskets were converted into daily amounts. In light of the differences in age, gender, and energy needs, family members do not receive the same share of the food available [25]. As a result, Adult Male Equivalent units (AMEs) were calculated for each member of the household instead of the per capita amount [25]. According to the FAO and WHO, AMEs represent the ratio between an adult male 18 to 30 years old with moderate physical activity and the energy requirement of a household member of that age. [26]. This study calculated the amount of total AME of the household based on the age and sex of household members. In order to determine the equivalent daily intake for an adult male of each food item, the amount of each food item was divided by the total number of AMEs in the household. In fact, we converted all members in this study to AME, therefore, this method would help us to do all calculations in the easiest way [27].

The FAO estimated waste percentages for each food group were used in the consumption step, "In steps from supply to consumption," to estimate the amount of food consumed, since this amount was purchased, received as gifts or donations, or produced by household members [28].

Energy intakes were then assessed using NUTRITIONIST IV software adapted to Iranian foods. Energy and nutrient contents of food items were analyzed using the USDA food composition table (FCT), and for traditional Iranian foods that were not provided by the USDA FCT, the Iranian FCT was used. Iranian FCT was added to Nutritionist IV, therefore for both foods which either supported or don't support USDA FCT, energy and nutrient were calculated. Food items were categorized into six categories based on the USDA food pyramid “Bread, cereal, rice, and pasta,” “Vegetables,” “Fruits,” “Dairy,” “Meat, poultry, fish, eggs, legumes, and nuts” and “Fats, Oils, Sugars, and Sweets” [29, 30]. Category of Food items were shown in Table 1 [31]. Furthermore, our food frequency questionnaire (FFQ) had 194 food items.

**Table 1:** Category of food items

Food groups	Food items
Bread, cereal, Breads, noodles, pasta, rice, barley, starch, popcorn, rice, and pasta cornflakes, wheat germ, bulgur	
Vegetables	Potatoes, cabbage, cauliflower, Brussels sprouts, broccoli, mixed vegetables, spinach, lettuce, cucumbers, eggplant, green peas, green beans, green peppers, turnips, corn, squash, celery, mushrooms, onions, garlic, tomatoes, carrots, olives
Fruits	Pears, apricots, cherries, apples, raisins or grapes, bananas, cantaloupe, watermelon, oranges, grapefruit, kiwi, grapefruits, strawberries, peaches, nectarines, tangerines, mulberries, plums, persimmons, pomegranates, lemons, pineapples, fresh figs, natural fruit juices, dried figs, dried dates, dried mulberries, other dried fruits
Milk, yogurt, and cheese	Milk, yogurt, flavored milk, cream, cheese, ice cream, dough
Meat, poultry, fish, eggs, legumes, and nuts	Processed meats, red meats, organ meats, poultry, fish, shrimp, eggs, beans, peas, lima beans, broad beans, lentils, soy, peanuts, almonds, pistachios, hazelnuts, roasted seeds, walnuts
Fats, oils, sugars, sweets	Hydrogenated fats, animal fats, mayonnaise, vegetable oils, chocolates, candies, tamarisk, honey, artificial juice, jam, jelly, cookies, cakes, confections

#### Other factors

According to our previous systematic review study [27], we detected factors which influence in household food consumption. Among these potential factors, we used factors which are available in our dataset. Therefore, this study modeled food consumption outcomes as a function of household head age, socioeconomic variables, household size, place of residence, and household head sex (which were available in the present study).

A socioeconomic status (SES) variable was extracted using Principal Component Analysis (PCA). Education, occupational status, and income status of households were used to define SES in this study. To determine socioeconomic status, one variable for household head occupation (managed, professional, technician, associate professional, clerk, services worker, skilled agricultural, forestry, and fishing worker, craft worker, plant and machine operator and assembly, elementary occupations, armed forces occupations), household income, education level, and house area were included in PCA. As a socioeconomic variable, we chose the factor with the highest eigenvalue. In this factor, all imputed items scored at least 0.40. Overall, 54.3% of the variance was explained by this factor. Households were classified based on their socioeconomic status.

As above mentioned, we used PCA to extract the SES variable and then categorized it into quartiles using IBM SPSS statistics version 26. The quartiles were interpreted as follows: SES 1 represented the category of the poorest, SES 2 represented the lower middle class, SES 3 represented the upper middle class, and SES 4 represented the highest category. We considered SES 1 and SES 4 as the lowest and highest quartiles in our study population, respectively. Therefore, SES 1 and SES 4 represent the lowest and highest levels of socioeconomic status, respectively, based on our study population.

In this study trained and experienced questionnaire conducted face-to-face interviews with the heads of households and asked them a series of questions about their household for the purposes of the study (reference [24] provides more comprehensive and detailed information).

### Statistical analysis

A dichotomization of baseline characteristics was conducted for two years (2019 and 2020). Data were presented as mean ± standard deviation for continuous variables, and as percentages for discrete variables. Categorical variables were compared using chi-square tests, and continuous variables were analyzed using independent-samples T-tests and Mann–Whitney tests. Version 26 of SPSS software was used for the statistical analysis. Levels of statistical significance were set at 0.05.

## Results

A total of 75,885 households were included in our study, 48.3% (*n* = 36,681) of these lived in urban areas. The participants were classified into two groups (urban and rural areas). Their characteristics are shown in Table 2. In each group, the participants were divided into 4 categories in terms of SES SES 1 and SES 4 were the lowest and highest quartiles in our study population, respectively. Thus, SES 1 and SES 4 had the lowest and the highest status (based on our population), respectively. The majority of households (83.5%) were male and 14.3% were female. Most of our participants were illiterate. Further, marital status was divided into 4 categories: married, widow, single and divorced. The significant difference was observed in literacy (*P*-value < 0.001) among urban population at two time points. Also, among rural population there were significant differences in age (*P*-value < 0.001) and material status (*P*-value = 0.013) before and after COVID-19 pandemic (Table 2).

**Table 2:** General characteristics of participants

Variables	Urban			Rural		
	2019 mean ± SD or number (%)	2020 mean ± SD or number (%)	*P-value*	2019 mean ± SD or number (%)	2020 mean ± SD or number (%)	*P-value*
Age	52.14 ± 16.25	52.26 ± 15.75	0.459	50.93 ± 14.96	51.50 ± 14.80	< 0.001
SES category			1.00			1.00
SES 1	4607 (25.0)	4562 (25.0)		4974 (25.0)	4826 (25.0)	
SES 2	4607 (25.0)	4563 (25.0)		4974 (25.0)	4827 (25.0)	
SES 3	4608 (25.0)	4563 (25.0)		4975 (25.0)	4827 (25.0)	
SES 4	4607 (25.0)	4562 (25.0)		4974 (25.0)	4826 (25.0)	
Literacy			< 0.001			0.199
Uneducated	12,384 (67.2)	12,726 (69.7)		16,566 (83.3)	16,167 (83.7)	
Primary or higher education	6045 (32.8)	5524 (30.3)		3331 (16.7)	3139 (16.3)	
Marital status			0.364			0.013
Married	15,385 (83.5)	15,151 (83.0)		16,926 (85.1)	16,221 (84.0)	
Widow	2629 (14.3)	2640 (14.5)		2345 (11.8)	2421 (12.5)	
Single	192 (1.0)	215 (1.2)		350 (1.8)	399 (2.1)	
Divorced	223 (1.2)	244 (1.3)		276 (1.4)	265 (1.4)	

Data were presented as N (%) for categorical variables and mean ± SD for continuous variables

*SES* Socioeconomic status

The significant level is considered as *P-value* < 0.05

Table 3 demonstrated the changes in food group consumption (% of energy) before and after COVID-19 pandemic. In the urban area, the consumption of grains group (%Δ = 2.09%) and vegetables group (%Δ = 4.38%) significantly increased. In contrast, the consumption of fruits group (%Δ = − 6.93%), dairy (%Δ = − 4.22%), meat group (%Δ = − 1.36%), and fats and sweets group (%Δ = − 3.04%) significantly decreased, although the consumption meat on its own (not meat group) (%Δ = 9.88%) significantly increased. In the rural area, the consumption of all food groups significantly decreased except meat group (%Δ = 6.22%) and vegetable group (%Δ = 7.21%). Table 4 presents the comparison of energy and macronutrient consumption between urban and rural in 2019 and 2020. Remarkably, in the urban area, we found a significant decrease in the consumption of energy (%Δ = − 3.67%) and fat (%Δ = − 1.40%). In contrast, we found a significant increase in the consumption of protein (%Δ = 0.38%) and carbohydrate (%Δ = 0.56%). In rural areas, we found a significant decrease in the consumption of carbohydrate (%Δ = − 0.71%). However, we found a significant increase in the consumption of protein (%Δ = 0.52%) and fat (%Δ = 1.24%).

**Table 3:** The comparison of food group (% of energy) consumption between urban and rural at two time points (2019 and 2020)

Variables	Urban				Rural			
	2019 mean ± SD	2020 mean ± SD	Difference (%)	*P-value*	2019 mean ± SD	2020 mean ± SD	Difference (%)	*P-value*
1. Grain Group	54.16 ± 13.67	55.29 ± 13.51	2.09	< 0.001	53.16 ± 13.48	52.68 ± 13.23	− 0.90	0.001
Bread	33.41 ± 19.12	34.08 ± 19.66	2.01	0.001	38.29 ± 15.60	38.04 ± 15.68	− 0.65	0.122
Cereal	8.10 ± 18.02	8.56 ± 18.33	5.68	0.021	1.31 ± 6.80	1.60 ± 7.13	22.14	< 0.001
Rice and pasta	12.64 ± 10.20	12.64 ± 10.47	0.00	0.999	13.54 ± 11.07	13.03 ± 10.80	− 3.77	< 0.001
2. Vegetables Group	4.57 ± 2.71	4.77 ± 2.66	4.38	< 0.001	4.99 ± 2.93	5.35 ± 2.71	7.21	< 0.001
Starchy roots	1.53 ± 1.34	1.72 ± 1.31	12.42	< 0.001	1.66 ± 1.17	1.81 ± 1.22	9.04	< 0.001
Cruciferous vegetables	0.03 ± 0.12	0.03 ± 0.13	0.00	0.190	0.05 ± 0.15	0.05 ± 0.13	0.00	0.836
Leafy green vegetables	0.26 ± 0.31	0.25 ± 0.30	− 3.85	0.013	0.35 ± 0.35	0.32 ± 0.32	− 8.57	< 0.001
Other vegetables	2.77 ± 1.95	2.79 ± 1.88	0.72	0.340	2.98 ± 2.26	3.21 ± 1.96	7.72	< 0.001
3. Fruits Group	2.74 ± 2.64	2.55 ± 2.59	− 6.93	< 0.001	3.53 ± 2.85	3.19 ± 2.54	− 9.63	< 0.001
Fresh fruits	2.58 ± 2.55	2.36 ± 2.48	− 8.53	< 0.001	3.37 ± 2.71	2.95 ± 2.39	− 12.46	< 0.001
Dried fruits	0.16 ± 0.57	0.18 ± 0.64	12.50	0.006	0.16 ± 0.68	0.23 ± 0.68	43.75	< 0.001
4. Dairy	4.74 ± 3.54	4.54 ± 3.35	− 4.22	< 0.001	4.99 ± 3.44	5.05 ± 3.06	1.20	0.063
5. Meat Group	11.07 ± 6.07	10.92 ± 5.88	− 1.36	0.016	11.58 ± 5.96	12.30 ± 5.79	6.22	< 0.001
Red meat	0.81 ± 2.78	0.89 ± 2.76	9.88	0.005	0.78 ± 1.82	0.90 ± 2.01	15.38	< 0.001
Processed meat	0.12 ± 0.43	0.12 ± 0.40	0.00	0.192	0.10 ± 0.47	0.17 ± 0.48	70.00	< 0.001
Poultry	4.31 ± 2.76	4.28 ± 2.70	− 0.70	0.182	4.84 ± 3.00	4.54 ± 2.70	− 6.20	< 0.001
Fish	0.28 ± 0.86	0.27 ± 0.75	− 3.57	0.530	0.38 ± 0.89	0.37 ± 0.85	− 2.63	0.448
Eggs	1.88 ± 1.59	1.95 ± 1.55	3.72	< 0.001	2.47 ± 1.73	2.55 ± 1.55	3.24	< 0.001
Legumes	3.21 ± 3.87	2.85 ± 3.49	− 11.21	< 0.001	2.41 ± 3.62	2.92 ± 3.45	21.16	< 0.001
Nuts	0.43 ± 1.42	0.53 ± 1.56	23.26	< 0.001	0.57 ± 1.87	0.89 ± 1.97	56.14	< 0.001
6. Fats, oils, sugars, and sweets	23.01 ± 10.12	22.31 ± 9.92	− 3.04	< 0.001	22.54 ± 10.27	22.21 ± 9.51	− 1.46	0.001
Hydrogenated fats*	6.86 ± 9.56	5.34 ± 8.83	− 22.16	< 0.001	4.94 ± 8.79	3.92 ± 7.61	− 20.65	< 0.001
Vegetable oils	7.66 ± 7.63	8.86 ± 7.90	15.67	< 0.001	9.37 ± 8.19	10.18 ± 7.81	8.64	< 0.001
Sugar	7.60 ± 5.39	7.31 ± 4.97	− 3.82	< 0.001	7.03 ± 5.68	6.99 ± 4.92	− 0.57	0.446
Sweets desserts	0.87 ± 1.66	0.79 ± 1.66	− 9.20	< 0.001	1.18 ± 2.24	1.10 ± 1.99	− 6.78	< 0.001

*Hydrogenated fats (also called trans-fatty acids) are manufactured fats created during a process called hydrogenation whereby hydrogen units are added to polyunsaturated fatty acids to prevent them from becoming rancid and to keep them solid at room temperature

Statistics are expressed as mean ± SD

*P-value* was reported by t-test for a comparison of food group consumption (% of energy) between urban and rural at two time points (2019 and 2020s)

The significant level is considered as *P-value* < 0.05

**Table 4:** The comparison of energy and macronutrients consumption between urban and rural at two time points (2019 and 2020)

Variables	Urban				Rural			
	2019 mean ± SD	2020 mean ± SD	Difference (%)	*P-value*	2019 mean ± SD	2020 mean ± SD	Differences(%)	*P-value*
Total energy (kcl)	2956.95 ± 1143.68	2848.51 ± 1088	− 3.67	< 0.001	2664.16 ± 1033.56	2683.69 ± 1008.58	0.73	0.068
Protein*	13.16 ± 2.29	13.21 ± 2.21	0.38	0.036	13.34 ± 2.33	13.41 ± 2.15	0.52	0.007
Carbohydrate*	60.87 ± 7.83	61.21 ± 7.72	0.56	< 0.001	60.67 ± 7.69	60.24 ± 7.34	− 0.71	< 0.001
Fat*	27.90 ± 8.45	27.51 ± 8.35	− 1.40	< 0.001	28.27 ± 8.19	28.62 ± 7.77	1.24	< 0.001

*Presented as % of total energy

Statistics are expressed as mean ± SD

*P-value* was reported by t-test for a comparison of energy and macronutrients consumption between urban and rural at two time points (2019 and 2020)

The significant level is considered as *P-value* < 0.05

The comparison of food group (% of energy) consumption between the category of SES score is summarized in Table 5 in two time points. The consumption of grains groups significantly increased in SES 3 and 4 (%Δ = 0.73%, %Δ = 1.21%; respectively), as regards the consumption of rice and pasta in SES 3 and 4 (%Δ = − 2.70%, %Δ = − 2.80%; respectively) decreased. In all of SES categories, the consumption of vegetable group (%Δ = 10.57%, %Δ = 5.08%, %Δ = 4.18%, %Δ = 3.43%; respectively) and meats group (%Δ = 2.53%, %Δ = 3.89%, %Δ = 2.18%, %Δ = 1.81%; respectively) significantly increased. In contrast, the consumption of fruits group and fats, oils, sugars, and sweets group decreased in all of SES categories. In addition, changes in dairy consumption were only significant in SES 3 (%Δ = − 2.59%). Table 6 shows the changes in energy and macronutrients consumption between the category of SES score before and after the COVID-19 pandemic. The changes in total energy intake were significantly decrease only in SES 1 (Δ% = − 1.96%), SES 2 (Δ% = − 1.70%) and SES 4 (Δ% = − 1.37%). In addition, changes in macronutrient consumption were significant only in protein consumption in SES 2 (Δ% = 0.69%).

**Table 5:** The comparison of food group (% of energy) consumption between category of SES score at two time points (2019 and 2020)

Variables	SES 1				SES 2				SES 3				SES 4			
	2019 mean ± SD	2020 mean ± SD	Difference(%)	*P-value*	2019 mean ± SD	2020 mean ± SD	Difference(%)	*P-value*	2019 mean ± SD	2020 mean ± SD	Difference(%)	*P-value*	2019 mean ± SD	2020 mean ± SD	Difference(%)	*P-value*
1. Grain Group	56.16 ± 14.65	56.19 ± 14.76	0.05	0.875	54.80 ± 13.10	54.96 ± 12.82	0.29	0.392	53.24 ± 12.71	53.63 ± 12.58	0.73	0.043	50.52 ± 13.23	51.13 ± 13.01	1.21	0.002
Bread	39.84 ± 19.91	39.82 ± 20.17	− 0.05	0.939	36.58 ± 17.64	36.62 ± 17.72	0.11	0.906	34.83 ± 16.52	35.21 ± 17.03	1.09	0.124	32.70 ± 15.20	33.06 ± 15.60	1.10	0.123
Cereal	5.04 ± 15.50	5.27 ± 15.76	4.56	0.332	5.14 ± 14.73	5.40 ± 14.82	5.06	0.240	4.70 ± 13.65	5.06 ± 13.83	7.66	0.076	3.52 ± 11.23	4.19 ± 12.16	19.03	< 0.001
Rice and pasta	11.27 ± 11.06	11.09 ± 11.08	− 1.60	0.306	13.06 ± 10.18	12.94 ± 10.14	− 0.92	0.412	13.71 ± 10.30	13.34 ± 10.30	− 2.70	0.017	14.28 ± 10.89	13.88 ± 10.84	− 2.80	0.014
2. Vegetables Group	4.73 ± 2.90	5.23 ± 2.82	10.57	< 0.001	4.72 ± 2.75	4.96 ± 2.65	5.08	< 0.001	4.78 ± 2.92	4.98 ± 2.68	4.18	< 0.001	4.95 ± 2.76	5.12 ± 2.65	3.43	< 0.001
Starchy roots	1.68 ± 1.35	1.88 ± 1.33	11.90	< 0.001	1.59 ± 1.21	1.77 ± 1.30	11.32	< 0.001	1.58 ± 1.37	1.73 ± 1.26	9.49	< 0.001	1.55 ± 1.09	1.70 ± 1.55	9.68	< 0.001
Cruciferous vegetables	0.02 ± 0.11	0.03 ± 0.14	50.00	0.179	0.04 ± 0.14	0.04 ± 0.12	0.00	0.020	0.04 ± 0.14	0.04 ± 0.12	0.00	0.701	0.05 ± 0.16	0.05 ± 0.13	0.00	0.218
Leafy green vegetables	0.25 ± 0.34	0.23 ± 0.32	− 8.00	< 0.001	0.29 ± 0.33	0.28 ± 0.31	− 3.45	0.035	0.32 ± 0.32	0.30 ± 0.30	− 6.25	< 0.001	0.36 ± 0.34	0.34 ± 0.32	− 5.56	< 0.001
Other vegetables	2.79 ± 2.13	3.11 ± 1.99	11.47	< 0.001	2.83 ± 2.05	2.90 ± 1.89	2.47	0.011	2.86 ± 2.18	2.94 ± 1.92	2.80	0.005	3.03 ± 2.11	3.07 ± 1.93	1.32	0.170
3. Fruits Group	2.49 ± 2.51	2.26 ± 2.31	− 9.24	< 0.001	2.86 ± 2.48	2.65 ± 2.31	− 7.34	< 0.001	3.25 ± 2.62	2.93 ± 2.44	− 9.85	< 0.001	3.98 ± 3.21	3.62 ± 3.02	− 9.05	< 0.001
Fresh fruits	2.35 ± 2.38	2.06 ± 2.14	− 12.34	< 0.001	2.72 ± 2.40	2.46 ± 2.16	− 9.56	< 0.001	3.08 ± 2.50	2.73 ± 2.33	− 11.36	< 0.001	3.76 ± 3.10	3.37 ± 2.91	− 10.37	< 0.001
Dried fruits	0.14 ± 0.64	0.19 ± 0.71	35.71	< 0.001	0.13 ± 0.50	0.19 ± 0.62	46.15	< 0.001	0.16 ± 0.63	0.19 ± 0.58	18.75	< 0.001	0.21 ± 0.71	0.25 ± 0.79	19.05	0.001
4. Dairy	4.32 ± 3.59	4.31 ± 3.17	− 0.23	0.862	4.64 ± 3.33	4.61 ± 3.23	− 0.65	0.524	5.01 ± 3.39	4.88 ± 3.12	− 2.59	0.007	5.46 ± 3.56	5.36 ± 3.25	− 1.83	0.075
5. Meat Group	10.27 ± 5.94	10.53 ± 5.80	2.53	0.004	10.81 ± 5.76	11.23 ± 5.64	3.89	< 0.001	11.46 ± 5.68	11.71 ± 5.62	2.18	0.003	12.74 ± 6.400	12.97 ± 6.15	1.81	0.014
Red meat	0.44 ± 1.86	0.50 ± 1.67	13.64	0.031	0.62 ± 2.12	0.68 ± 1.90	9.68	0.058	0.79 ± 2.13	0.92 ± 2.36	16.46	< 0.001	1.29 ± 2.97	1.47 ± 3.17	13.95	< 0.001
Processed meat	0.07 ± 0.36	0.09 ± 0.38	28.57	< 0.001	0.11 ± 0.43	0.16 ± 0.47	45.45	< 0.001	0.14 ± 0.51	0.16 ± 0.46	14.29	0.008	0.14 ± 0.49	0.16 ± 0.46	14.29	0.001
Poultry	4.67 ± 3.36	4.50 ± 3.15	− 3.64	0.001	4.44 ± 2.68	4.32 ± 2.63	− 2.70	0.004	4.54 ± 2.72	4.37 ± 2.49	− 3.74	< 0.001	4.69 ± 2.80	4.46 ± 2.53	− 4.90	< 0.001
Fish	0.19 ± 0.67	0.22 ± 0.75	15.79	0.003	0.26 ± 0.93	0.27 ± 0.73	3.85	0.533	0.34 ± 0.83	0.32 ± 0.79	− 5.88	0.085	0.51 ± 1.00	0.47 ± 0.90	− 7.84	0.002
Eggs	2.17 ± 1.89	2.26 ± 1.75	4.15	0.001	2.16 ± 1.68	2.23 ± 1.52	3.24	0.004	2.17 ± 1.57	2.25 ± 1.51	3.69	< 0.001	2.23 ± 1.61	2.28 ± 1.54	2.24	0.022
Legumes	2.46 ± 3.70	2.56 ± 3.40	4.07	0.062	2.81 ± 3.80	2.96 ± 3.61	5.34	0.007	2.94 ± 3.77	2.97 ± 3.40	1.02	0.507	2.96 ± 3.76	3.02 ± 3.44	2.03	0.268
Nuts	0.24 ± 1.19	0.37 ± 1.42	54.17	< 0.001	0.37 ± 1.32	0.57 ± 1.53	54.05	< 0.001	0.51 ± 1.47	0.69 ± 1.70	35.29	< 0.001	0.89 ± 2.36	1.08 ± 2.28	21.35	< 0.001
6. Fats, oils, sugars, and sweets	22.71 ± 11.56	22.17 ± 11.12	− 2.38	0.002	22.77 ± 9.91	22.15 ± 9.45	− 2.72	< 0.001	22.75 ± 9.56	22.41 ± 9.10	− 1.49	0.014	22.84 ± 9.71	22.29 ± 9.12	− 2.41	< 0.001
Hydrogenated fats*	6.53 ± 10.49	4.67 ± 9.22	− 28.48	< 0.001	6.45 ± 9.33	5.01 ± 8.44	− 22.33	< 0.001	5.76 ± 8.73	4.91 ± 8.10	− 14.76	< 0.001	4.76 ± 8.12	3.86 ± 7.12	− 18.91	< 0.001
Vegetable oils	8.20 ± 8.75	9.90 ± 8.94	20.73	< 0.001	8.12 ± 7.89	9.26 ± 7.81	14.04	< 0.001	8.62 ± 7.65	9.25 ± 7.49	7.31	< 0.001	9.22 ± 7.54	9.76 ± 7.23	5.86	< 0.001
Sugar	7.39 ± 5.98	7.02 ± 5.98	− 5.01	< 0.001	7.30 ± 5.43	7.01 ± 4.84	− 3.97	< 0.001	7.18 ± 5.29	7.21 ± 4.62	0.42	0.714	7.37 ± 5.49	7.33 ± 4.85	− 0.54	0.584
Sweets desserts	0.58 ± 1.74	0.56 ± 1.69	− 3.45	0.622	0.88 ± 1.73	0.85 ± 1.71	− 3.41	0.303	1.17 ± 2.03	1.03 ± 1.800	− 11.97	< 0.001	1.47 ± 2.27	1.32 ± 2.07	− 10.20	< 0.001

*Hydrogenated fats (also called trans-fatty acids) are manufactured fats created during a process called hydrogenation whereby hydrogen units are added to polyunsaturated fatty acids to prevent them from becoming rancid and to keep them solid at room temperature

Statistics are expressed as mean ± SD

*SES* Socioeconomic status

*P-value* was reported by t-test for a comparison of food group (% of energy) consumption between category of SES score at two time points (2019 and 2020)

The significant level is considered as *P-value* < 0.05

**Table 6:** The comparison of energy and macronutrients consumption between categories of SES score at two time points (2019 and 2020)

Variables	SES 1				SES 2				SES 3				SES 4			
	2019 mean ± SD	2020 mean ± SD	Difference (%)	*P-value*	2019 mean ± SD	2020 mean ± SD	Difference(%)	*P-value*	2019 mean ± SD	2020 mean ± SD	Difference (%)	*P-value*	2019 mean ± SD	2020 mean ± SD	Difference (%)	*P-value*
Total energy (kcl)	2986.08 ± 1214.82	2927.45 ± 1174.43	− 1.96	0.001	2787.04 ± 1089.94	2739.75 ± 1038.52	− 1.70	0.003	2715.81 ± 1021.67	2691.65 ± 979.34	− 0.89	0.106	2742.96 ± 1041.98	2705.35 ± 993.20	− 1.37	0.014
Protein*	13.13 ± 2.53	13.18 ± 2.29	0.38	0.229	13.10 ± 2.23	13.19 ± 2.11	0.69	0.006	13.24 ± 2.12	13.28 ± 2.10	0.30	0.179	13.54 ± 2.34	13.59 ± 2.21	0.37	0.144
Carbohydrate*	61.22 ± 8.53	61.03 ± 8.35	− 0.31	0.151	61.10 ± 7.54	60.99 ± 7.28	− 0.18	0.307	60.70 ± 7.30	60.69 ± 7.03	− 0.02	0.897	60.08 ± 7.60	60.17 ± 7.45	0.15	0.430
Fat*	27.75 ± 9.32	27.87 ± 9.12	0.43	0.384	27.87 ± 8.15	27.89 ± 7.87	0.07	0.861	28.16 ± 7.82	28.14 ± 7.55	− 0.07	0.910	28.56 ± 7.94	28.41 ± 7.72	− 0.53	0.188

*Presented as % of total energy

Statistics are expressed as mean ± SD

*SES* Socioeconomic status

*P-*value was reborted by t-test for a comparison of energy and macronutrients consumption between categories of SES score at two time points (2019 and 2020)

The significant level is considered as *P*-value < 0.05

## Discussion

The present study has examined the changes between food groups, energy and macronutrients consumption before and after COVID-19 pandemic among urban and rural population and also in different SES categories.

In urban area, the consumption of grains and vegetables had increased, however, the consumption of fruits, dairy, meat group, and fats and sweets were decreased. In addition, we observed increase in consumption of protein and carbohydrate, however decrease in consumption of energy and fat. In rural area, the consumption of all of food groups was decreased except meat and vegetables. Moreover, our results indicated an increase in consumption of protein and fat, in contrast, a decrease in consumption of carbohydrates.

Since the unexpected outbreak of the COVID-19 pandemic, food shopping and consumption habits among consumers have been disrupted [32–34]. Chung-Cheng Yang et al. showed that the COVID-19 pandemic has increased the overall food consumption of the Chinese people due to a strong economy, a high level of population savings, and positive actions by food companies to improve consumption [35]. In contrast, some studies show that food consumption may have decreased due to declining incomes, loss of businesses and use of more of the income for medical care [11, 36]. In addition, during the pandemic, due to the reduction of food production and the disruption of the food supply chain, food groups faced shortages and increased prices [37]. This shock in low- and middle-income countries leads to the inability to afford healthy and nutritious food group [38]. Furthermore, almost all countries implemented health strategies including quarantine and social distancing to delay disease transmission [4]. These health strategies reduced the labor force (to harvest and transfer food from farm to distributors), and thus increased delivery times [6, 39, 40]. As well, consumers do not have sufficient access to healthy food because of the limited amount of travel and working hours in stores [6, 11]. Further, consumers are reluctant to go to markets and supermarkets due to the possibility of contracting COVID-19 in stores [41]. As well as, households' access to food decreased, especially among low-income groups and rural area due to job loss or reduced household income during the pandemic [11, 42].

In addition to the COVID-19 pandemic, a study in Iran showed that the economic recession at the same time as COVID-19 can lead to food insecurity due to financial losses and price increases [43]. Iran, even before COVID-19, has been affected by macroeconomic problems (such as inflation and high exchange rates) and a decrease in per capita income. These factors can lead to an increase in production costs and, in turn, an increase in the price of all kinds of food. In addition, the excess supply of some food items caused a decrease in prices and financial damage for producers, which can threaten our food security [43]. Besides the economic recession, environmental restrictions (such as climate change, severe soil erosion, deforestation, etc.) that are expanding in Iran can cause problems by disrupting the agricultural sector in the economy and food security [43–46]. Hence, the COVID-19 pandemic, besides directly affecting food consumption, can worsen this situation by disrupting the production cycle and ignoring environmental protection policies. [43, 44]. Therefore, COVID-19 can be one of the main influencing factors in the household food basket.

Rural areas of Iran are at risk of food insecurity due to social inequalities and limited access to health care [47]. Hence, we decided to divide our population into two urban and rural groups. Regarding to our results, people in rural areas tended to increase their consumption of fresh food during the quarantine, especially vegetables and meat, which is inconsistent with Meike Janssen's et al. findings based on a cross-sectional online survey of 2680 residents of Denmark, Germany and Slovenia [16]. This could be because people in rural areas used their livestock to prepare meat and had gardens in their fields to supply their vegetables [48, 49]. On the other hand, fruit consumption decreased due to the possible reduction of labor force for harvesting and storage, and the increase in the price of agricultural pesticides [40, 50]. In contrast, people in urban areas tended to increase vegetable and grains consumption, which is consistent with the findings of Celia Rodríguez-Pérez et al. [51] and Bahareh Nikooyeh et al. [22]. This may be because people believe that consuming vegetables is good for health and boosts the immune system, which in turn leads to more consumption [52]. Also, grains are one of the cheap and staple foods that are the main source of energy in Iranians' diet, which causes more consumption [22].

Based on our findings, the COVID-19 pandemic could have different effects on energy and macronutrient intake, which could be due to changes in food consumption patterns as a result of the pandemic [53]. Since meat and grains are sources of protein and carbohydrates in the diet of Iranians [22, 54], protein consumption increased due to the increase in meat consumption in urban and rural areas, and changes in carbohydrate consumption were due to changes in grain consumption in urban and rural areas. Also, the increase in the consumption of meat group in rural areas can lead to an increase in consumption of fat.

Regarding the comparison of food groups, energy and macronutrient consumption between category of SES score at two time points (before and after COVID-19), the consumption of grains increased in SES 3 and 4. In all of SES categories, the consumption of vegetables and meats increased, in contrast the consumption of fruits, fats, oils, sugars, and sweets decreased. In addition, the total energy intake decreased only in SES 1, SES 2 and SES 4.

According to a study in Iran, SES of households was the most important determinant of food insecurity in poor areas [55]. Also, the coincidence of the COVID-19 epidemic with the economic crisis could aggravate poverty and food insecurity [43, 55]. Hence, we decided to stratify our population by SES. This study found that all SES groups could suffer negative consequences in food group consumption from the COVID-19 pandemic. The possible reason for the increase in grains consumption in SES 3 and SES 4 is that the income of the upper SES category has decreased compared to before the COVID-19 [11], while grains consumption remained low in the low SES category before COVID-19, so we did not witness any significant changes. Since meat is a rich source of zinc and is effective in boosting the immune response [56], it can be a good food choice for people in the face of an epidemic. Therefore, this is an explanation for the increase in meat consumption in all SES categories. Our study reported an increase in vegetable consumption and a decrease in fruits, fats, oils, sugars, and sweets consumption in all SES categories, while some studies reported a decrease [34, 57] and others an increase [1, 58] in fruit and vegetable consumption. Also, a study in Iran reported an increase in fruits and vegetables in the adult population, which was against our results [21]. This can be due to WHO and government recommendations to increase the consumption of legumes, fruits and vegetables and reduce fats, oils, sugars, and sweets during the pandemic [59, 60]. These recommendations were also presented in Iran [21], which led to an increase in interest in the consumption of vegetables due to their positive effectiveness in COVID-19 [52], while this opinion was not about fruits. Furthermore, the decrease in fruit consumption in our study can be due to disruption in agricultural productivity, reduction in labor force and increase in price [5, 61], which is the result of the possible effects of COVID-19. In contrast, another study in Tehran, Iran reported a decrease in fruit and vegetable consumption, which could be due to food factories closing due to the virus outbreak. As a result, it led to a decrease in food availability and an increase in food prices [62].

Our findings showed that the pandemic could have different effects on energy and macronutrient intake in SES categories, which could be due to changes in food consumption patterns as a result of the pandemic [53]. Also, studies reported that food consumption decreased during the pandemic [16, 53], which could explain the decrease in energy in different SES categories. In addition, a study in Iran, like our study, showed that the situation of food consumption was affected by the epidemic of COVID-19, which could be due to the limitation of transportation, disruption in the cycle of food production and supply [47].

The present study had some limitations. Firstly, asking about participants' past consumption depended on memory which was related to recall bias. Secondly, cross-sectional design of our study was another important limitation. Thirdly, lack of investigation of food waste was a further limitation. Despite these limitations, our study had some points of strength, including a high sample size and consideration of urban and rural areas and SES separately for food consumption. In addition, the coverage of energy and macronutrient intake can be a strong point to differentiate the study from other studies. Also, our data were based on the total amount of food purchased, food items received, and any food produced by household members.

## Conclusion

In general, among the population of urban and rural areas as well as in different SES categories, people tended to increase their consumption of meat and fresh food, especially vegetable groups. In contrast, we observed a decrease in the consumption of fruit, fat, and sweets groups. In addition, studies have shown that food consumption has declined during the pandemic, which could explain the decline in energy in different categories of SES. Since fruits and vegetables are good sources of vitamins, antioxidants, and phytochemicals, they can be effective in boosting the immune system. Hence, reducing the consumption of these substances can be considered a risk factor for COVID-19 diseases, which can disrupt the body's immunity against the virus. Consequently, the impact of COVID-19 on nutrient intake and predicting health outcomes at the public health level are important.

## Data Availability

Datasets used in the current study are available from the corresponding author upon reasonable request.

## Abbreviations

COVID-19: Coronavirus disease 2019
SES: Socioeconomic status
SCI: Statistical Centre of Iran
HIES: Households income and expenditure survey
SARS-CoV-2: Severe acute respiratory syndrome coronavirus 2
AMEs: Adult male equivalent units

## References

[1] Ruiz-Roso MB, de Carvalho PP, Mantilla-Escalante DC, Ulloa N, Brun P, Acevedo-Correa D (2020). Covid-19 confinement and changes of adolescent's dietary trends in Italy, Spain, Chile, Colombia and Brazil. Nutrients.

[2] Mignogna C, Costanzo S, Ghulam A, Cerletti C, Donati MB, de Gaetano G (2021). Impact of nationwide lockdowns resulting from the first wave of the COVID-19 pandemic on food intake, eating behaviours and diet quality: a systematic review. Adv Nutr (Bethesda, Md).

[3] Wang J, Yeoh EK, Yung TKC, Wong MCS, Dong D, Chen X (2021). Change in eating habits and physical activities before and during the COVID-19 pandemic in Hong Kong: a cross-sectional study via random telephone survey. J Int Soc Sports Nutr.

[4] Sepúlveda-Loyola W, Rodríguez-Sánchez I, Pérez-Rodríguez P, Ganz F, Torralba R, Oliveira DV (2020). Impact of social isolation due to COVID-19 on health in older people: mental and physical effects and recommendations. J Nutr Health Aging.

[5] Di Renzo L, Gualtieri P, Pivari F, Soldati L, Attinà A, Cinelli G (2020). Eating habits and lifestyle changes during COVID-19 lockdown: an Italian survey. J Transl Med.

[6] Aday S, Aday MS (2020). Impact of COVID-19 on the food supply chain. Food Qual Saf.

[7] Mattioli AV, Ballerini Puviani M, Nasi M, Farinetti A (2020). COVID-19 pandemic: the effects of quarantine on cardiovascular risk. Eur J Clin Nutr.

[8] Brooks SK, Webster RK, Smith LE, Woodland L, Wessely S, Greenberg N (2020). The psychological impact of quarantine and how to reduce it: rapid review of the evidence. Lancet (London, England).

[9] Anton SD, Miller PM (2005). Do negative emotions predict alcohol consumption, saturated fat intake, and physical activity in older adults?. Behav Modif.

[10] Macht M (2008). How emotions affect eating: a five-way model. Appetite.

[11] Carducci B, Keats EC, Ruel M, Haddad L, Osendarp SJM, Bhutta ZA (2021). Food systems, diets and nutrition in the wake of COVID-19. Nature Food.

[12] Benker B (2021). Stockpiling as resilience: defending and contextualising extra food procurement during lockdown. Appetite.

[13] Wang E, An N, Gao Z, Kiprop E, Geng X (2020). Consumer food stockpiling behavior and willingness to pay for food reserves in COVID-19. Food Secur.

[14] Swinnen J, McDermott J (2020). COVID-19 and global food security. EuroChoices.

[15] Mun H, So ES (2022). Changes in physical activity, healthy diet, and sleeping time during the COVID-19 pandemic in South Korea. Nutrients.

[16] Janssen M, Chang BPI, Hristov H, Pravst I, Profeta A, Millard J (2021). Changes in food consumption during the COVID-19 Pandemic: analysis of consumer survey data from the first lockdown period in Denmark, Germany, and Slovenia. Front Nutr.

[17] Profeta A, Siddiqui SA, Smetana S, Hossaini SM, Heinz V, Kircher C (2021). The impact of Corona pandemic on consumer's food consumption. J Consumer Protect Food Saf.

[18] Mattioli AV, Sciomer S, Cocchi C, Maffei S, Gallina S (2020). Quarantine during COVID-19 outbreak: Changes in diet and physical activity increase the risk of cardiovascular disease. Nutr Metab Cardiovasc Dis.

[19] Mishra P, Parveen R, Bajpai R, Samim M, Agarwal NB (2021). Impact of cardiovascular diseases on severity of COVID-19 patients: a systematic review. Ann Acad Med Singapore.

[20] Poly TN, Islam MM, Yang HC, Lin MC, Jian W-S, Hsu M-H (2021). Obesity and mortality among patients diagnosed with COVID-19: a systematic review and meta-analysis. Front Med.

[21] Akbarzadeh M, Barati-Boldaji R, Mohsenpour MA, Ferns GA, Jalali M, Mosallanezhad Z (2021). Did Iranians change their eating behavior following COVID-19 outbreak?. J Res Med Sci.

[22] Nikooyeh B, Rabiei S, Amini M, Ghodsi D, Rasekhi H, Doustmohammadian A (2022). COVID-19 epidemic lockdown-induced changes of cereals and animal protein foods consumption of Iran population: the first nationwide survey. J Health Popul Nutr.

[23] Rasekhi H, Rabiei S, Amini M, Ghodsi D, Doustmohammadian A, Nikooyeh B (2021). COVID-19 epidemic-induced changes of dietary intake of Iran population during lockdown period: the study protocol (study protocol article). Nutr Food Sci Res.

[24] Statistical Center of Iran Household, Expenditure and Income [Available from: https://www.amar.org.ir/english/Metadata/Statistical-Survey/Household-Expenditure-and-Income (accessed January 2023).

[25] Weisell R, Dop MC (2012). The adult male equivalent concept and its application to Household Consumption and Expenditures Surveys (HCES). Food Nutr Bull.

[26] University UN, Organization WH. Human Energy Requirements: Report of a Joint FAO/WHO/UNU Expert Consultation: Rome, 17–24 October 2001: Food & Agriculture Org.; 2004.

[27] Eini-Zinab H, Sobhani SR, Rezazadeh A (2021). Designing a healthy, low-cost and environmentally sustainable food basket: an optimisation study. Public Health Nutr.

[28] Gustafsson J, Cederberg C, Sonesson U, Emanuelsson A. The methodology of the FAO study: Global Food Losses and Food Waste-extent, causes and prevention”-FAO, 2011. SIK Institutet för livsmedel och bioteknik; 2013.

[29] Welsh SO, Davis C, Shaw A. USDA's food guide: background and development. Miscellaneous publication (USA). 1993.

[30] Health UDo, Services H. Dietary guidelines for Americans 2005. http://www.health.gov/dietaryguidelines/dga2005/document/defaulthtm. 2005.

[31] Sobhani SR, Eini-Zinab H, Rezazadeh A (2021). Assessing the changes in iranian household food basket using national household budget and expenditure survey data, 1991–2017. Int J Prev Med.

[32] Yi-Chi Chang Y, Wu PL, Chiou WB (1982). Thoughts of social distancing experiences affect food intake and hypothetical binge eating: Implications for people in home quarantine during COVID-19. Soc Sci Med.

[33] Jia P, Liu L, Xie X, Yuan C, Chen H, Guo B (2021). Changes in dietary patterns among youths in China during COVID-19 epidemic: The COVID-19 impact on lifestyle change survey (COINLICS). Appetite.

[34] Bracale R, Vaccaro CM (2020). Changes in food choice following restrictive measures due to Covid-19. Nutr Metab Cardiovasc Dis.

[35] Yang CC, Chen YS, Chen J (2022). The impact of the COVID-19 pandemic on food consumption behavior: based on the perspective of accounting data of chinese food enterprises and economic theory. Nutrients.

[36] Vaidheeswaran S, Karmugilan MK. Consumer buying behaviour on healthcare products and medical devices during COVID-19 pandemic period-a new spotlight. Nveo-Natural Volatiles Essent Oils J. NVEO. 2021:9861–72.

[37] Falkendal T, Otto C, Schewe J, Jägermeyr J, Konar M, Kummu M (2021). Grain export restrictions during COVID-19 risk food insecurity in many low-and middle-income countries. Nat Food.

[38] Laborde D, Herforth A, Headey D, de Pee S (2021). COVID-19 pandemic leads to greater depth of unaffordability of healthy and nutrient-adequate diets in low-and middle-income countries. Nat. Food.

[39] Garnett P, Doherty B, Heron T (2020). Vulnerability of the United Kingdom’s food supply chains exposed by COVID-19. Nat. Food.

[40] Wang Y, Wang J, Wang X. COVID-19, supply chain disruption and China’s hog market: a dynamic analysis. China Agricultural Economic Review. 2020.

[41] FAO. Impacts of coronavirus on food security and nutrition in Asia and the Pacific: building more resilient food systems. FAO Bangkok, Thailand; 2020.

[42] Ghanbari Movahed R, Maleki Fard F, Gholamrezai S, Pakravan-Charvadeh MR (2022). The Impact of COVID-19 Pandemic on Food Security and Food Diversity of Iranian Rural Households. Front Public Health.

[43] Rad AK, Shamshiri RR, Azarm H, Balasundram SK, Sultan M (2021). Effects of the COVID-19 pandemic on food security and agriculture in Iran: a survey. Sustainability.

[44] Barani N, Karami A, Borazjani MA (2020). The impact of climatic changes on total horticultural production and food security in agro-ecological zones of Iran. J Water Clim Change.

[45] Nations U. Deserts and Fight against Desertification [Available from: https://www.un.org/development/desa/dpad/publication/world-economic-situation-and-prospects-2019/.

[46] Fuentes R, Galeotti M, Lanza A, Manzano B (2020). COVID-19 and climate change: a tale of two global problems. Sustainability.

[47] Ghanbari Movahed R, Maleki Fard F, Gholamrezai S, Pakravan-Charvadeh MR. The Impact of COVID-19 Pandemic on Food Security and Food Diversity of Iranian Rural Households. Frontiers in Public Health. 2022;10. 10.3389/fpubh.2022.862043 PMC9008508 35433601

[48] Zulu SS, Ngidi M, Ojo T, Hlatshwayo SI (2022). Determinants of consumers’ acceptance of indigenous leafy vegetables in Limpopo and Mpumalanga provinces of South Africa. J Ethnic Foods.

[49] Smith J, Sones K, Grace D, MacMillan S, Tarawali S, Herrero M (2013). Beyond milk, meat, and eggs: role of livestock in food and nutrition security. Anim Front.

[50] Boughton D, Goeb J, Lambrecht I, Headey D, Takeshima H, Mahrt K (2021). Impacts of COVID-19 on agricultural production and food systems in late transforming Southeast Asia: the case of Myanmar. Agric Syst.

[51] Rodríguez-Pérez C, Molina-Montes E, Verardo V, Artacho R, García-Villanova B, Guerra-Hernández EJ (2020). Changes in Dietary Behaviours during the COVID-19 Outbreak Confinement in the Spanish COVIDiet Study. Nutrients.

[52] Emanuel AS, McCully SN, Gallagher KM, Updegraff JA (2012). Theory of Planned Behavior explains gender difference in fruit and vegetable consumption. Appetite.

[53] Han X, Guo Y, Xue P, Wang X, Zhu W (2022). Impacts of COVID-19 on nutritional intake in rural China: panel data evidence. Nutrients.

[54] Islami F, Malekshah AF, Kimiagar M, Pourshams A, Wakefield J, Goglani G (2009). Patterns of food and nutrient consumption in northern Iran, a high-risk area for esophageal cancer. Nutr Cancer.

[55] Joulaei H, Keshani P, Foroozanfar Z, Afrashteh S, Hosseinkhani Z, Mohsenpour MA (2023). Food insecurity status and its contributing factors in slums’ dwellers of southwest Iran, 2021: a cross-sectional study. Arch Public Health.

[56] Laubertová L, Koňariková K, Gbelcová H, Ďuračková Z, Muchová J, Garaiova I (2017). Fish oil emulsion supplementation might improve quality of life of diabetic patients due to its antioxidant and anti-inflammatory properties. Nutr Res (New York, NY).

[57] Sidor A, Rzymski P (2020). Dietary choices and habits during COVID-19 lockdown: experience from Poland. Nutrients.

[58] Carroll N, Sadowski A, Laila A, Hruska V, Nixon M, Ma DWL (2020). The Impact of COVID-19 on health behavior, stress, financial and food security among middle to high income canadian families with young children. Nutrients.

[59] EMRO W. Nutrition advice for adults during the COVID-19 outbreak 2021 [Available from: http://www.emro.who.int/nutrition/news/nutrition-advice-for-adults-during-the-covid-19-outbreak.html.

[60] Organization WH. Coronavirus disease (COVID-19): food safety and nutrition. 2020.

[61] Mehta V. The impact of COVID-19 on the dietary habits of middle-class population in Mulund, Mumbai, India. AIJR Preprints. 2020;1(10).

[62] Pakravan-Charvadeh MR, Mohammadi-Nasrabadi F, Gholamrezai S, Vatanparast H, Flora C, Nabavi-Pelesaraei A (2021). The short-term effects of COVID-19 outbreak on dietary diversity and food security status of Iranian households (A case study in Tehran province). J Clean Prod.

